# Molecular Characterization of Representative CPV-2c Isolates and Establishment of VP2-Targeted Nanobody-Based Immunodetection Tools

**DOI:** 10.3390/ani16091402

**Published:** 2026-05-03

**Authors:** Liangkai Liu, Maohua Xia, Chengyao Hou, Danyu Chen, Chengyao Li, Xinggui Chen, Qinyuan Chu, Yue Sun, Shujun Liu, Yuqing Li, Hanlin Wang, Yan Zhu, Mengfang Yang, Hongning Wang, Caiwu Li, Xin Yang

**Affiliations:** 1 Animal Disease Prevention and Green Development Key Laboratory of Sichuan Province, Key Laboratory of Bio-Resource and Eco-Environment of Ministry of Education, College of Life Sciences, Sichuan University, Chengdu 610064, China; 2 Beijing Zoo Administration Office, Beijing Key Laboratory of Captive Wildlife Technologies, Beijing 100044, China; 3 China Conservation and Research Center for the Giant Panda, Key Laboratory of SFGA on the Giant Panda, Chengdu 610057, China

**Keywords:** canine parvovirus, virus isolation, nanobody, immunodetection

## Abstract

Although canine parvovirus (CPV) vaccination has been widely implemented, CPV continues to circulate in dog populations and poses a potential cross-species transmission risk to wildlife, including giant pandas. Recent increases in CPV-2c detection in China highlight the need for molecular surveillance and standardized immunoreagents for diagnosis and epitope mapping. This study aimed to isolate a representative CPV-2c strain from China and develop VP2-targeted nanobody-based recognition molecules to support antigen monitoring and detection optimization. Canine and giant panda samples were collected in Sichuan Province, and CPV was isolated in F81 cells, followed by VP2 gene sequencing and phylogenetic analysis. A secretion expression system in Bacillus subtilis was established to produce VP2-targeting nanobodies, and a canine Fc-fused format of Nb10 (Nb10-Fc) was constructed. Immunoreactivity was evaluated via immunoassays, and structural modeling and molecular docking were performed to predict binding interfaces. The results showed that CPV-2c was the dominant genotype in Sichuan, with CPV L4 being a representative strain that exhibited 100% identity in VP2 with a giant panda-derived CPV-2c strain. Nb10 and Nb10-Fc demonstrated strong reactivity in Western blotting and immunofluorescence assays. The Fc-fusion improved detection sensitivity, offering potential in vivo application benefits. This study provides a standardized VP2-specific nanobody and molecular system for CPV-2c surveillance, antigenic studies, and diagnostic optimization.

## Simple Summary

This study focuses on canine parvovirus (CPV), especially the CPV-2c subtype that is increasingly spreading in China and may infect not only dogs but also wildlife like giant pandas. The researchers isolated a representative strain (CPV L4) and analyzed its genetic characteristics, confirming that CPV-2c is the dominant type in the region. They also developed special detection tools using nanobodies (small antibodies) targeting the VP2 protein of the virus. One nanobody (Nb10) and its improved version (Nb10-Fc) showed a strong ability to detect the virus in laboratory tests, with the Fc-fusion enhancing sensitivity and stability. Overall, the study provides useful tools and data for monitoring CPV-2c, improving diagnosis, and supporting future vaccine and antiviral research.

## 1. Introduction

Canine parvovirus (CPV) is an important enteric pathogen of canids, primarily infecting puppies and causing acute hemorrhagic enteritis with high morbidity and mortality [1]. CPV belongs to the family Parvoviridae, genus Protoparvovirus, and is classified as Protoparvovirus carnivoran 1 [2]. The virus is non-enveloped with a stable capsid structure [3], exhibits strong environmental resistance, and relies on host–cell DNA synthesis-related mechanisms for replication. Therefore, it preferentially proliferates in rapidly dividing cells (such as intestinal crypt epithelial cells and hematopoietic cells), leading to typical clinical manifestations associated with intestinal injury. In addition to domestic dogs, CPV can infect multiple carnivore species, including the giant panda, indicating its potential for cross-species transmission [4]. In scenarios where companion animals may come into contact with wildlife, the spillover risk of canine-origin pathogens is of concern for endangered species conservation and ecological security.

The CPV genome is approximately 5.2 kb single-stranded DNA [5]. The VP2 protein is the major structural protein of the CPV capsid and plays a central role in receptor binding, host range determination, and immune recognition; it is also the major antigenic target of neutralizing antibodies [6]. Amino acid substitutions on the VP2 surface can alter capsid surface charge distribution and spatial conformation, thereby affecting the exposure of antigenic determinants and the antibody recognition spectrum. Therefore, VP2 variation not only determines genotype classification but is also closely related to antigenic difference assessment. Antibodies targeting VP2 can serve as important tools for virus identification and antigenic characterization, particularly in Western blotting and immunofluorescence assays, supporting studies on the antigenic profiles of circulating strains [7].

Despite the long-term and widespread use of vaccines, CPV continues to circulate in dog populations and shows marked genetic diversity and lineage turnover. In recent years, CPV-2c strains have been increasingly detected in China and worldwide [8] and were first identified in Italy in 2000 [9], gradually becoming one of the major circulating genotypes. In high-density urban environments, dog aggregation, increased mobility, and persistent environmental contamination provide conditions for long-term transmission and accumulation of mutations [1]. Meanwhile, in zoos, rescue and breeding centers, and surrounding areas, potential overlaps between dog activity and the living environments of wildlife (including giant pandas) make CPV surveillance meaningful beyond dog populations alone. Therefore, within an epidemiological surveillance framework, establishing analytical systems that reflect the genetic and antigenic characteristics of current circulating strains is crucial for risk assessment and optimization of prevention and control strategies. CPV genotyping not only serves epidemiological description but also supports strain tracing, evolutionary inference, and antigenic profile evaluation. Currently, the classification of variants such as CPV-2a, CPV-2b, and CPV-2c is mainly based on characteristic amino acid differences in the VP2 protein (or VP2 gene), among which the VP2 residue 426 is a classic genotyping marker used to distinguish CPV-2a (426N), CPV-2b (426D), and CPV-2c (426E) [10]. In addition to residue 426, substitutions at multiple sites on VP2 are commonly used for molecular epidemiology and lineage identification, reflecting continuous evolution during natural transmission. Thus, phylogenetic analysis and mutation statistics based on VP2 sequences have become core approaches for analyzing the molecular characteristics of circulating strains and supporting subsequent antigenic evaluation.

Nanobodies (VHH) are derived from the variable region of naturally occurring heavy-chain antibodies in camelids and have advantages such as small molecular weight, high thermal stability, and ease of expression and engineering [11]. In recent years, nanobodies have attracted extensive attention in virology research and immunodetection. Due to good tissue penetration and easy conjugation with fluorescent or radioactive labels, nanobodies have been widely used for molecular imaging and in vivo tracing [12], enabling rapid target localization and dynamic monitoring. Their high specificity also makes them suitable for Western blotting [13], immunofluorescence, and ELISA [14], and they can serve as important molecular probes for epitope mapping and antigen structure studies. In antiviral therapy research, nanobodies can also be engineered as neutralizing molecules or fused with Fc fragments to construct multivalent formats [15], thereby expanding their applications. Therefore, VP2-targeting nanobodies not only facilitate immunological identification and antigenic characterization of circulating CPV strains but also provide a tool basis for subsequent development of diagnostic reagents and antibody-based products.

In this study, CPV was isolated and identified from canine clinical samples in China, and the VP2 gene of the obtained isolates was sequenced for genetic characterization and phylogenetic analysis to evaluate their relationships and genotyping features relative to domestic and international strains. Meanwhile, based on VP2 nanobody sequences preserved in our laboratory [16], nanobodies and their Fc-fused formats were constructed and expressed, and their applicability in immunological detection platforms such as Western blotting, immunofluorescence, and ELISA was systematically evaluated. Combined with computer-aided structural modeling, molecular docking, and dynamic analysis, the potential binding interface and key interacting residues between the nanobody and VP2 were predicted, providing a theoretical basis for antigenic characterization of VP2 in circulating CPV strains and the development of molecular tools for detection.

## 2. Materials and Methods

### 2.1. Sample Collection and Cells

Clinical samples suspected of CPV infection were collected from pet hospitals in Chengdu, Sichuan Province, China, and the Ya’an Giant Panda Protection Center. A total of 32 clinical samples were collected, including 30 canine samples and 2 giant panda samples. Samples were collected between January and June 2025, and the animals included in this study presented with clinical symptoms such as diarrhea. Fecal swabs were collected from symptomatic dogs and giant pandas. F81 cells (Cyagen, Guangzhou, China) were cultured in RPMI 1640 medium (Gibco, Shanghai, China) supplemented with 10% fetal bovine serum (Cellmax, Beijing, China).

### 2.2. Virus Isolation and Identification

Fecal swab samples were subjected to repeated freeze–thaw cycles and centrifuged at 12,000× *g* for 10 min. The supernatants were filtered through a 0.22 µm membrane and aliquoted for subsequent virus isolation. F81 cells were used for virus amplification. Because CPV can efficiently replicate only in actively proliferating cells, F81 cells were cultured to approximately 70–80% confluence prior to inoculation to ensure cells were in the growth phase. Sample supernatants were added to cell monolayers and adsorbed for 2 h at 37 °C. After adsorption, the inoculum was discarded, and RPMI 1640 maintenance medium containing 2% FBS was added. During serial passaging, viral replication and cytopathic effects (CPEs) were carefully monitored.

### 2.3. PCR

Viral DNA was extracted from clinical materials using a genomic DNA extraction kit (Tiangen, Beijing, China) according to the manufacturer’s instructions. To obtain the full-length VP2 gene sequence, two primer pairs were used to amplify the full VP2 fragments (F1: CCAGTATTAACTGATACACCAGA, R1: CAACCTCAGCTGGTCTCATAAT; F2: CATGTAGACTAACACACATGG, R2: CCATATAACAAACCTTCTAAATCCT). In this study, the primers used for PCR amplification were designed based on conserved regions of the VP2 gene and were validated prior to use. The expected size of the amplification product was 392 bp. PCR products were examined by 1.5% agarose gel electrophoresis, recovered, and sequenced. The full-length VP2 gene sequence was obtained by assembly. To verify successful virus isolation, DNA was extracted from infected cells after five blind passages in F81 cells, and PCR identification was performed using specific primers targeting a conserved region of VP2 (F: ACTCAGCCACCAACTAAAG, R: GGTAAGCCCAATGCTCTAT). Products were analyzed by 1.5% agarose gel electrophoresis to determine whether CPV was successfully isolated.

### 2.4. Transmission Electron Microscopy (TEM)

After low-speed centrifugation to remove cell debris, clarified supernatants were loaded onto a pre-prepared sucrose gradient for ultracentrifugation to enrich viral particles. The visible enrichment band was collected, diluted with PBS, and subjected to ultrafiltration to remove sucrose. The final concentrate was resuspended in a small volume of PBS for TEM observation. A 5–10 µL aliquot of enriched virus suspension was applied onto carbon-coated copper grids and adsorbed at room temperature for 5–10 min. Excess liquid was removed, and grids were negatively stained with 1–2% phosphotungstic acid (PTA, pH ~7.0) for 30–60 s, air-dried, and observed under a transmission electron microscope to analyze particle morphology and size.

### 2.5. Western Blot

Western blotting was used to detect VP2 protein expression in virus-infected cells, evaluate nanobody recognition of VP2, and identify tagged nanobody expression products. After lysis, samples were centrifuged at 12,000× *g* for 10 min. Supernatants were separated by SDS-PAGE and transferred onto PVDF membranes, which were blocked with 5% skim milk at room temperature. For VP2 detection, membranes were incubated with a mouse anti-CPV VP2 primary antibody (Bioss, Beijing, China, bsm-49051M) followed by HRP-conjugated goat anti-mouse IgG (H + L) (Beyotime, Shanghai, China, A0216). For nanobody binding validation, Nb10 or Nb10-Fc was used as the binding antibody. Nb10 signals were detected using an anti-His primary antibody (Proteintech, Wuhan, China, 66005-1-Ig) and HRP-conjugated goat anti-mouse IgG (H + L), whereas Nb10-Fc was detected using rabbit anti-dog IgG-HRP (Solarbio, Beijing, China, SE236). For nanobody expression identification, mouse anti-His or anti-HA primary antibodies (Proteintech, Wuhan, China, 66006-2-Ig) and HRP-conjugated goat anti-mouse IgG (H + L) were used. Membranes were washed with TBST, developed with chemiluminescent substrate, and imaged.

### 2.6. Immunofluorescence Assay

Immunofluorescence assays were performed to detect VP2 expression in CPV-infected cells and to validate Nb10 and Nb10-Fc recognition of CPV L4 at the cellular level. CPV L4, a representative CPV-2c strain isolated in this study, was selected for subsequent analyses. At 36 h post-inoculation, F81 cells were fixed with 4% paraformaldehyde at room temperature for 15 min and blocked with immunofluorescence blocking buffer (Beyotime, Shanghai, China, P0260) for 15 min. For VP2 detection, cells were incubated with mouse anti-CPV VP2 primary antibody (Abcam, Shanghai, China, ab140431) at 37 °C for 1 h, followed by AF488-labeled goat anti-mouse secondary antibody (Beyotime, Shanghai, China, A1089-100 μL) at 37 °C for 45 min. For Nb10 validation, cells were incubated with diluted Nb10 at 37 °C for 1 h, followed by mouse anti-His antibody (Proteintech, Wuhan, China, 66005-1-Ig) for 1 h and AF488-labeled goat anti-mouse secondary antibody for 45 min. For Nb10-Fc validation, cells were incubated with diluted Nb10-Fc at 37 °C for 1 h, followed by rabbit anti-dog IgG–FITC (Solarbio, Beijing, China, SF236) at 37 °C for 45 min. Cells were washed three times with PBS between steps, nuclei were stained with DAPI for 5 min, and images were acquired using a fluorescence microscope.

### 2.7. Preparation of Standard Curve

A plasmid containing the target gene was used as a template for 10-fold serial dilutions from 10^0^ to 10^6^. Cq values were determined by quantitative PCR for each dilution. The standard curve was generated by plotting copy number against Cq value to calculate the relationship between them. The standard curve was established for absolute quantification of viral genome copies and was used in the viral growth kinetics assay. The standard equation was log_10_(copies/µL) = −0.3452 × Cq + 12.4, R^2^ = 0.9958.

### 2.8. Viral Growth Kinetics

Replication kinetics of CPV L4 were evaluated by copy number determination. F81 cell monolayers were infected at MOI = 0.1. Culture supernatants and cells were collected at 0, 12, 24, 36, 48, 60, and 72 h post-infection, subjected to repeated freeze–thaw cycles, and used as virus samples. Viral copy numbers were determined by absolute quantitative PCR. Data are presented as mean ± standard deviation and used to plot growth curves.

### 2.9. Phylogenetic and Recombination Analysis

Phylogenetic analysis of CPV isolates was performed based on VP2 amino acid sequences. The sequences include the positive sequences obtained in this study and the reference sequences downloaded from GenBank. Sequence alignment was performed using MEGA X. A maximum-likelihood phylogenetic tree was constructed in MEGA X and evaluated by bootstrap analysis with 1000 replicates. The tree was visualized and annotated using iTOL. To assess potential recombination events, the Sequence Demarcation Tool was used for recombination analysis of VP2 amino acid sequences by calculating pairwise identity and displaying similarity matrices to identify abnormally high-homology fragments or recombination signals. To systematically analyze molecular variation in VP2, VP2 amino acid sequences of multiple CPV isolates obtained in this study were aligned with the prototype strain CPV-2 (GenBank accession no. M19296.1). Amino acid substitutions relative to the prototype were counted, mutation frequencies were calculated, and mutation distributions on VP2 were visualized. In addition, VP2 sequences from multiple CPV-2c strains reported to infect giant pandas were selected as references and aligned with VP2 sequences of isolates obtained in this study to identify amino acid differences. All sequences used, along with their GenBank accession numbers, are listed in Supplementary Table S1.

### 2.10. Expression and Characterization of Nanobodies

The nucleotide sequences of nanobodies Nb10, Nb12, Nb43, Nb66, and Nb76 were derived from previous laboratory work [16]. Based on these sequences, nanobodies were constructed, secreted, and identified. For secretory nanobody production, nanobody genes were cloned into the Bacillus subtilis secretory expression vector PHT-43 and fused with a 6 × His tag at the C-terminus for purification and detection. Recombinant plasmids were introduced into competent Bacillus subtilis cells by electroporation to obtain positive recombinant strains. Positive strains were expanded and induced under established conditions. After induction, cultures were centrifuged to remove bacterial cells, and supernatants were collected as the source of secreted proteins. His-tagged nanobodies in the supernatants were purified using Ni-NTA affinity chromatography and eluted. Eluates were further buffer-exchanged and concentrated using 3 kDa cutoff ultrafiltration tubes. Purified products were analyzed by SDS-PAGE and Coomassie Brilliant Blue staining to evaluate expression and purification efficiency based on specific bands at the expected molecular weight.

### 2.11. Expression of Nb10-Fc

To obtain the Nb10-Fc fusion protein, a eukaryotic secretory expression vector was constructed. The pCMV vector was used as a backbone, with high-level expression driven by the CMV promoter. An IgK signal peptide was introduced at the N-terminus to mediate secretion into the culture supernatant, and an HA tag was fused for expression identification. The Nb10 coding sequence was linked with the canine IgG Fc fragment in the same open reading frame to generate the Nb10-Fc expression plasmid. 293T cells were cultured in DMEM supplemented with 10% FBS to ~70–80% confluence and transiently transfected using polyethyleneimine (PEI). After transfection, the medium was replaced with fresh complete medium, and supernatants were collected at 48 h post-transfection. Supernatants were clarified by low-speed centrifugation and filtration and used as secreted Nb10-Fc samples for Western blotting, ELISA, and immunofluorescence assays.

### 2.12. Indirect ELISA for Nanobody Binding to VP2

Indirect ELISA was used to evaluate binding reactivity between nanobodies and VP2 protein. High-binding 96-well plates were coated with recombinant VP2 protein and incubated overnight at 4 °C. The next day, the coating solution was discarded, and the plates were washed with PBST and blocked with 5% skim milk at room temperature for 1 h. Different nanobody solutions were added and incubated at room temperature. After washing, HRP-conjugated secondary antibodies corresponding to nanobody tags were added and incubated at room temperature. After washing, the TMB substrate was added, and the reaction was stopped with the stop solution. Absorbance was read at 450 nm. Negative control wells were included, and at least three independent experiments were performed.

### 2.13. Computer-Aided Structural Modeling and Docking

AlphaFold3 was used to predict the 3D structures of nanobody Nb10, fusion protein Nb10-Fc, and CPV L4 VP2, and the model with the highest pLDDT score was selected for analysis. Global protein–protein docking was performed using the HDOCKlite v1.1 server for Nb10–VP2 and Nb10-Fc–VP2 complexes. Binding free energy (ΔG) was calculated using the MM/GBSA method implemented on the HawkDock platform. Protein–protein interactions were analyzed using PLIP to identify key interfacial hydrogen bonds, salt bridges, and π–cation interactions. Docking conformations and key interfaces were visualized using PyMOL (PyMOL version 3.1.0, New York, NY, USA). 

### 2.14. De Novo Modeling by AlphaFold and Molecular Dynamics Simulations

The structures of the two complexes, CPV L4 VP2-Nb10 and CPV L4 VP2-Nb10-Fc, were modeled de novo using AlphaFold3. The best-ranked models were selected based on a weighted scoring function of predicted TM-score (pTM) and interface pTM (ipTM), calculated as 0.2 × pTM + 0.8 × ipTM. Visualizations of the resulting models were generated using PyMOL.

Molecular dynamics (MD) simulations were performed for both complexes using the GROMACS 2024.3, employing the CHARMM36m force field. Solvated systems were constructed in periodic boundary condition boxes of appropriate size, containing TIP3P water molecules and a neutralizing concentration of Na+ and Cl− ions. Prior to the production runs, a four-step equilibration protocol was implemented: energy minimization via the steepest descent algorithm, followed by two successive NVT heating phases and a final NPT equilibration stage. The production MD simulations were conducted with a time step of 2 fs for a total duration of 200 ns. To assess the structural stability and dynamic properties of the systems, Root Mean Square Deviation (RMSD), Root Mean Square Fluctuation (RMSF), hydrogen bond counts, Solvent-Accessible Surface Area (SASA), and Radius of Gyration (Rg) were analyzed using GROMACS utilities. Finally, the trajectory from the last 100 ns of each simulation was utilized to calculate the binding free energy between the antigen and the antibody/nanobody using the MM/PBSA method.

### 2.15. Statistical Analysis

Statistical significance was evaluated using Student’s t-test, one-way ANOVA, or two-way ANOVA depending on the experimental design. Significance thresholds were indicated as follows: * *p* < 0.05, ** *p* < 0.01, *** *p* < 0.001, and **** *p* < 0.0001. All analyses were performed using GraphPad Prism version 10.1.2 (GraphPad Software, San Diego, CA, USA). Data are presented as mean ± standard error of the mean (SEM).

## 3. Results

### 3.1. Isolation and Identification of CPV L4

**Figure 1:**
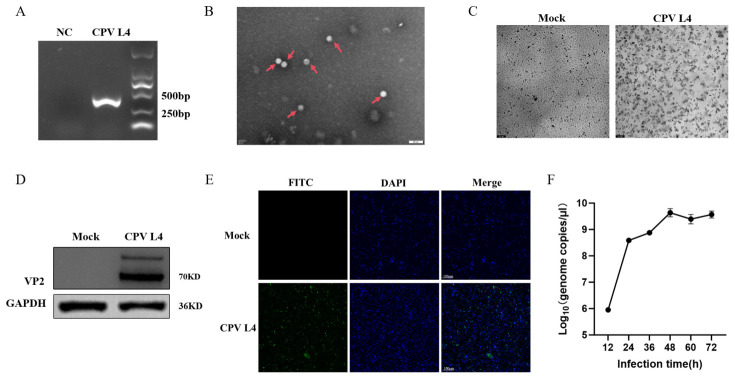
(**A**) PCR amplification results after five blind passages. (**B**) TEM observation of CPV L4. Arrows indicate spherical virus particles; scale bar, 50 nm. (**C**) Cytopathic effects of CPV L4 infection in F81 cells (MOI = 1). (**D**) Western blot analysis of VP2 expression in CPV L4-infected F81 cells (MOI = 1). Upper band was observed above the VP2 protein band, which may be caused by post-translational modification of the VP2 protein or incomplete denaturation. GAPDH served as an internal control. (**E**) Immunofluorescence detection of CPV L4-infected F81 cells (MOI = 1); scale bar, 100 µm. (**F**) Growth kinetics of CPV L4 in F81 cells (MOI = 0.1). Infected cells and supernatants were harvested every 12 h for 72 h. Viral genome copies are shown as log10(copies/µL). Data represent three independent experiments (triplicate each) and are presented as mean ± SEM.

The main clinical symptoms of dogs suspected of CPV infection included depression, anorexia, and bloody diarrhea. Eight positive samples were identified, corresponding to an overall positive rate of 25% (8/32). The positive rate in dogs was 26.67% (8/30), while no positives were detected in giant pandas (0/2). (Table 1). Full-length VP2 sequences of the eight positive samples were obtained and submitted to the NCBI GenBank database under accession numbers PX843424–PX843431, named CPV L1–L8. One CPV strain was successfully isolated from the eight positive samples and was designated CPV L4 in this study. To isolate and identify CPV, molecular, morphological, and cytological analyses were performed. Clarified swab supernatants were inoculated onto F81 cells and blindly passaged five times, followed by PCR amplification targeting VP2. A specific band of approximately 392 bp was obtained for CPV L4, while no band was detected in the negative control (Figure 1A), indicating successful isolation. Purified virus samples were negatively stained and observed by TEM, showing non-enveloped spherical particles with a diameter of approximately 20–25 nm (Figure 1B), consistent with CPV morphology. After inoculation onto F81 cells, infected cells gradually exhibited CPEs characterized by cell shrinkage and detachment, whereas control cells remained normal (Figure 1C). Western blot analysis detected a specific VP2 band in CPV L4-infected cells but not in controls (Figure 1D). Immunofluorescence assays showed VP2-specific fluorescence in infected cells but not in controls (Figure 1E), further confirming infection and protein expression.

In vitro growth kinetics showed that at MOI = 0.1, CPV L4 copy numbers increased over time and peaked at 72 h post-infection (Figure 1F). Collectively, these results demonstrate that a CPV strain, CPV L4, was successfully isolated and identified.

### 3.2. Phylogenetic Analysis

**Figure 2:**
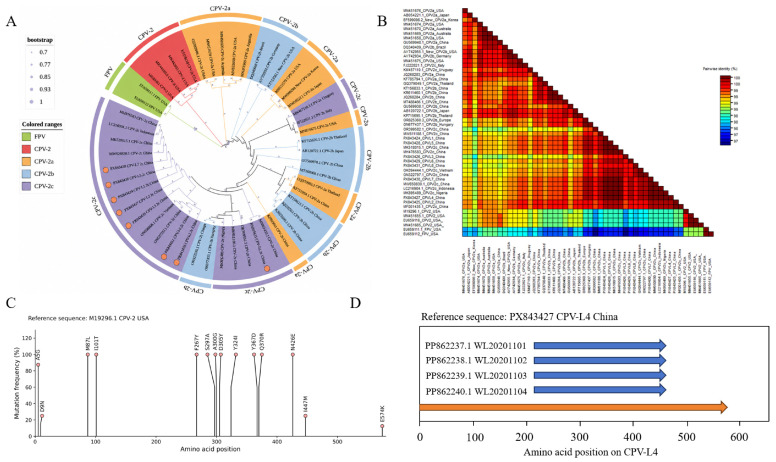
Genetic and evolutionary analysis of CPV VP2. (**A**) Maximum-likelihood phylogenetic tree of the CPV VP2 gene constructed using MEGA X with the JTT+G substitution model and 1000 bootstrap replicates. Orange circles indicate isolates CPV L1–L8. (**B**) Heatmap of amino acid similarity among CPV VP2 proteins. (**C**) VP2 amino acid mutation analysis of isolates CPV L1–L8 relative to prototype CPV-2. (**D**) Visual alignment of partial VP2 sequences between CPV L4 and giant panda-derived CPV-2c strains. The orange arrow indicates the full-length VP2 sequence of CPV L4, and blue arrows indicate reported partial VP2 sequences of four panda-infecting CPV-2c strains. Sequences are aligned by VP2 amino acid positions.

Based on VP2 gene sequences, a maximum-likelihood phylogenetic tree was constructed using MEGA X with the JTT+G substitution model and 1000 bootstrap replicates. The results showed that CPV L1–L8 clustered within the CPV-2c branch, indicating that all isolates obtained in this study belonged to CPV-2c, consistent with the currently circulating subtype in China (Figure 2A). Pairwise amino acid identity analysis showed differences in similarity among isolates and reported strains. CPV L1, L4, L5, L6, and L7 had the highest similarity (>99.8291%) with strain MW650830; CPV L2 showed 100% similarity with strain MF001435; CPV L3 showed the highest similarity (98.9744%) with strain OR399582; and CPV L8 showed 99.8291% similarity with strain ON322797 (Figure 2B). Using the prototype strain CPV-2 (M19296.1) as a reference, mutation analysis of VP2 amino acid sequences revealed multiple substitutions at positions including 267, 300, and 426, with different frequencies among isolates (Figure 2C).

To further compare CPV L4 with previously reported giant panda-infecting CPV-2c strains, VP2 amino acid sequences were aligned. CPV L4 VP2 fully covered the reported VP2 regions of panda-derived CPV-2c strains, and 100% sequence identity was observed in the overlapping region (Figure 2D).

### 3.3. Expression and Identification of Nanobodies

**Figure 3:**
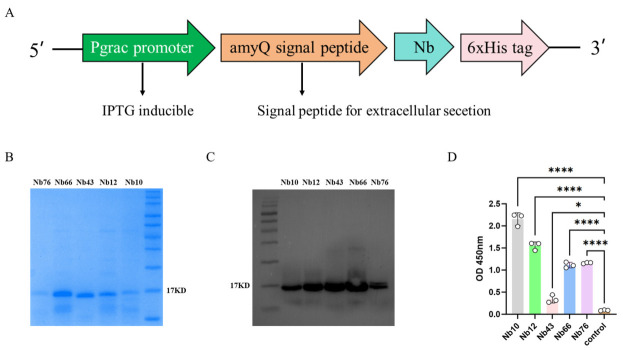
(**A**) Schematic of nanobody expression construct. Nanobody genes are expressed under the Pgrac promoter, fused with the amyQ signal peptide for secretion via the Sec pathway, and linked to a 6 × His tag for detection and purification. (**B**) SDS-PAGE analysis of five nanobodies in Bacillus subtilis culture supernatants. (**C**) Western blot identification of His tags for five nanobodies. (**D**) Indirect ELISA evaluation of binding reactivity between nanobodies and immunization VP2 protein; results are shown as OD450 values. * *p* < 0.05, **** *p* < 0.0001.

To obtain secreted nanobodies, an expression vector driven by the Pgrac promoter was constructed, with the amyQ signal peptide mediating secretion via the Sec pathway. Five nanobodies were fused with a 6 × His tag at the C-terminus for detection and purification (Figure 3A). SDS-PAGE analysis of purified nanobodies showed clear protein bands at the expected molecular weight (~17 kDa), indicating successful secretory expression in *Bacillus subtilis* (Figure 3B). Western blot identification of the His tag showed that all five nanobodies were specifically recognized by anti-His antibodies at the corresponding molecular weight, confirming the identity of the secreted proteins (Figure 3C). Indirect ELISA demonstrated differences in binding reactivity to immunization VP2 protein among nanobodies; Nb10 showed relatively higher OD450 values, while negative controls showed no signal (Figure 3D). Based on these results, Nb10 was selected for subsequent analyses.

### 3.4. Immunorecognition of CPV L4 by Nb10 and Structural Docking Analysis

**Figure 4:**
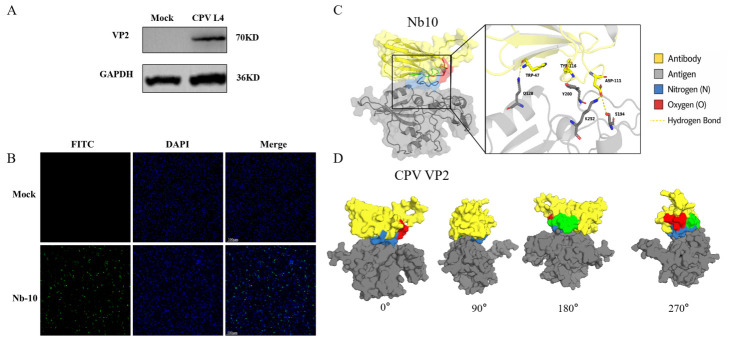
(**A**) Western blot validation of Nb10 recognition of VP2 in CPV L4-infected cells (MOI = 1); Nb10 served as the primary antibody. (**B**) Immunofluorescence detection of Nb10 recognition at the cellular level (MOI = 1); Nb10 served as the primary antibody; nuclei were stained with DAPI (blue). (**C**) Molecular docking model of Nb10 with CPV VP2 showing potential interaction sites. (**D**) Visualization of the Nb10–VP2 complex from different angles.

To evaluate the recognition ability of Nb10 against CPV L4, Western blotting was used to detect Nb10 binding to VP2 in CPV L4-infected cells. A specific band corresponding to VP2 was detected in infected samples, while no signal was observed in uninfected controls (Figure 4A), indicating that Nb10 specifically recognizes CPV L4 VP2 under denaturing conditions. Immunofluorescence assays further validated Nb10 recognition at the cellular level, showing clear specific green fluorescence in infected cells but not in controls (Figure 4B). These results demonstrate that Nb10 can specifically recognize CPV L4 antigen in cells and is suitable for immunofluorescence detection.

Based on this, 3D structural modeling and molecular docking analysis were performed for Nb10 and CPV VP2. Nb10 was predicted to bind a specific region on the VP2 surface and form a stable complex. Interface analysis indicated that interactions were mediated mainly by noncovalent forces such as hydrogen bonds (Figure 4C). Visualization from different angles showed stable binding of Nb10 to the VP2 surface with consistent interface location (Figure 4D).

### 3.5. Expression Identification, Immunorecognition, and Docking Analysis of Nb10-Fc

**Figure 5:**
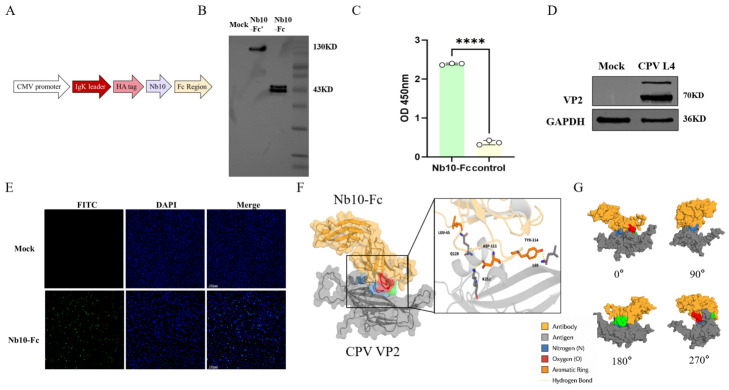
(**A**) Schematic of Nb10-Fc expression construct. (**B**) Western blot validation of Nb10-Fc expression. Nb10-Fc’ indicates the non-reducing condition result; Nb10-Fc indicates the reducing condition result. Nb10-Fc was detected under both conditions but showed different migration characteristics. (**C**) Indirect ELISA evaluation of Nb10-Fc binding to immunization VP2 protein (OD450). (**D**) Western blot validation of Nb10-Fc recognition of VP2 in CPV L4-infected cells (MOI = 1). Upper band was observed above the VP2 protein band, which may be caused by post-translational modification of the VP2 protein or incomplete denaturation. GAPDH served as an internal control. (**E**) Immunofluorescence detection of Nb10-Fc recognition in CPV L4-infected cells (MOI = 1). Specific green fluorescence was observed in infected cells; nuclei were stained with DAPI (blue). (**F**) Molecular docking model and interface analysis of Nb10-Fc with CPV VP2. (**G**) Visualization of the Nb10-Fc–VP2 complex from different angles. **** *p* < 0.0001.

To obtain Nb10-Fc, a eukaryotic expression vector driven by the CMV promoter was constructed, with an Igκ signal peptide mediating secretion, and expressed in 293T cells (Figure 5A). Western blot analysis confirmed expression of Nb10-Fc in culture supernatants. Under reducing conditions, a specific Nb10-Fc band was detected; under non-reducing conditions, Nb10-Fc showed a higher apparent molecular weight (Figure 5B), indicating different electrophoretic migration characteristics under different conditions. Indirect ELISA showed that Nb10-Fc produced clear binding signals in VP2-coated wells, whereas no signal was detected in controls (Figure 5C), indicating effective recognition of VP2. Western blot analysis further confirmed Nb10-Fc recognition of VP2 in CPV L4-infected cells (Figure 5D). Immunofluorescence assays showed obvious Nb10-Fc-specific fluorescence in infected cells but not in controls (Figure 5E), indicating that Nb10-Fc is suitable for cellular immunodetection.

In computer-aided structural analysis, molecular docking showed that Nb10-Fc binds a specific region on the VP2 surface and forms a stable complex (Figure 5F). Multi-angle visualization showed consistent binding interfaces across viewing angles (Figure 5G).

### 3.6. Molecular Dynamics Simulations

**Figure 6:**
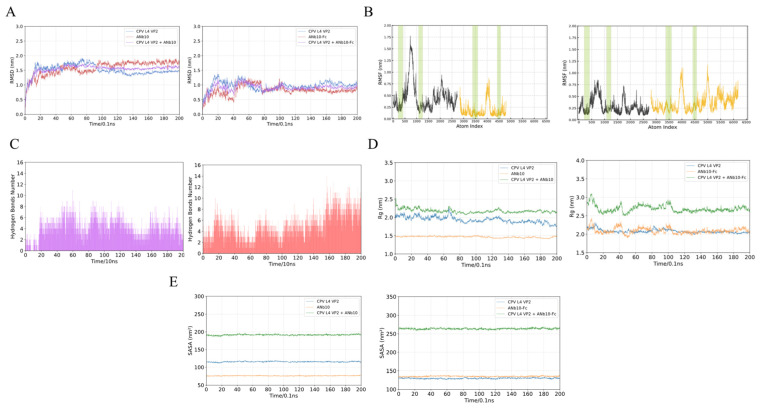
Dynamic property analyses of CPV L4 VP2–Nb10 (left) and CPV L4 VP2–Nb10-Fc (right) complexes. (**A**) RMSD curves; (**B**) RMSF curves (green shaded regions indicate interaction interfaces); (**C**) hydrogen bond numbers; (**D**) SASA curves; (**E**) radius of gyration (Rg) curves.

To evaluate antigen–antibody binding stability, molecular dynamics simulations were performed. Based on docking models (CPV L4 VP2–Nb10 and CPV L4 VP2–Nb10-Fc), 200 ns simulations were conducted for both complexes. RMSD curves showed that both complexes reached stability after ~100 ns (Figure 6A). RMSF analysis indicated low flexibility at the antigen–antibody interface, suggesting its key role in maintaining binding stability (Figure 6B). Hydrogen bond counts showed that stable binding was maintained mainly by 2–4 hydrogen bonds (Figure 6C). SASA curves indicated stable solvent-accessible surface areas for antibody, antigen, and complexes without major fluctuations (Figure 6D). Rg curves further indicated compact and stable conformations without large-scale structural rearrangements (Figure 6E).

MM/PBSA calculations showed binding free energies (ΔG) of −36.46 ± 0.42 kcal/mol and −35.11 ± 0.44 kcal/mol for CPV L4 VP2–Nb10 and CPV L4 VP2–Nb10-Fc, respectively (Table 2), indicating stable binding for both complexes. Residue energy contribution analysis identified multiple key residues involved in binding (Supplementary Figure S1), mainly located in antibody CDR regions and antigen flexible loops, consistent with RMSF results.

## 4. Discussion

Although CPV vaccines have been widely used in dog populations, CPV continues to circulate in many regions and poses a threat to wildlife such as the giant panda. Especially in high-density rearing and frequent contact environments such as urban settings, rapid viral evolution and mutation accumulation drive the emergence of multiple subtypes, potentially leading to insufficient antigenic coverage of vaccines based on the classical CPV-2 [17]. In recent years, the geographic range and detection numbers of CPV-2c in China have continued to increase [18], while vaccination has not significantly altered CPV epidemiological trends, suggesting that current immunoprevention systems may face challenges such as differences in immune protection. Therefore, continuous molecular surveillance of circulating strains and establishment of standardized antigen recognition and epitope mapping systems are of great significance for vaccine updating and risk assessment.

In this study, a CPV L4 strain was isolated from positive samples. Phylogenetic analysis showed that it was consistent with current circulating lineages in China and shared comparable identity with reported panda-derived sequences in key regions [19]. As a representative research strain, CPV L4 enhances the epidemiological relevance of our findings. Based on this, we utilized existing nanobody sequence resources in our laboratory to establish a *Bacillus subtilis* secretory expression system, yielding engineerable and scalable VP2 recognition tool molecules. Compared with traditional polyclonal/monoclonal antibodies, nanobodies have simpler structures, easier batch consistency control, and can be readily fused with tags or functional modules, making them suitable for standardized detection and epitope research platforms for circulating strains. To further enhance nanobody functionality, Nb10 was fused with the canine IgG Fc fragment (Nb10-Fc). This modification significantly improved molecular recognition: Fc-mediated natural dimerization enhanced avidity [20], thereby increasing antigen-binding strength and reducing dissociation rates, particularly suitable for scenarios with high antigen density or complex conformations. Importantly, Fc fusion also enhanced immunological functions. Through binding to Fcγ receptors, Nb10-Fc may mediate antibody-dependent cellular phagocytosis (ADCP) and antibody-dependent cellular cytotoxicity (ADCC) and may trigger complement responses, indicating potential viral clearance capacity [21]. In addition, the Fc fragment improved molecular stability: FcRn-mediated recycling prolongs in vivo half-life, enhancing serum stability and bioavailability [22], and improving signal stability across detection platforms (e.g., ELISA, Western blotting, and immunofluorescence) while reducing reliance on tag systems (e.g., His/HA) [23]. This modification not only improved detection sensitivity but also provided technical routes for purification and large-scale production. In addition, this study combines immune recognition experiments with structural bioinformatics strategies. Through structural modeling and molecular docking, the potential binding interface of Nb10-Fc with VP2 was predicted. It was found that Nb10-Fc tightly binds to VP2 through residues such as L69, K252, and Q128. K252 and L69 are highly conserved immune epitopes in CPV-2, while Q128, located on the surface of VP2, may represent a hotspot for immune escape mutations [24]. Mutations at these sites could significantly affect antibody binding and vaccine protection efficacy. This provides a theoretical basis for subsequent site mutation validation, escape mutation monitoring, and comparative analysis of epitope conservation across different viral lineages. Overall, the functional enhancement of Nb10-Fc provides a solid foundation for the development of diagnostic reagents and antibody-based formulations, advancing the establishment of a standardized antigen detection and epitope research platform aimed at epidemic strains.

This study has some limitations. First, VP2 sequence data were limited by regional and temporal coverage, making it difficult to fully reflect the genetic diversity and lineage turnover of CPV-2c. Future studies should expand sampling and sample size to improve representativeness. Secondly, Nb10-Fc requires in vivo evaluation of its serum stability and half-life, and its antiviral efficacy should be further investigated to support its translation into a functional molecule.

In conclusion, under the circumstances of CPV circulation and accelerated CPV-2c spread, this study used the epidemiologically representative CPV L4 as a research object and, combined with nanobody engineering strategies, established a standardized and scalable VP2 recognition tool molecular system. The Nb10-Fc fusion format enhanced recognition molecules in multivalent binding, detection signal stability, and methodological compatibility. Meanwhile, structural bioinformatics provided testable structural clues for potential binding interfaces, guiding subsequent epitope validation, escape mutation monitoring, and epitope conservation comparisons across lineages. Overall, this study provides a technical framework for continuous CPV-2c surveillance, antigenic mechanism studies, and diagnostic optimization in China and lays a foundation for future vaccine updating and antibody-based product development.

## 5. Conclusions

This study successfully isolated and identified a representative CPV-2c strain, CPV L4, and systematically analyzed its VP2 gene characteristics and genetic relationships with circulating domestic strains and a giant panda-derived CPV-2c strain, suggesting the continued circulation of CPV-2c and its potential risk of cross-species transmission. Furthermore, this study established a VP2-targeted nanobody-based immunorecognition system and demonstrated that Nb10 and its Fc-fused form (Nb10-Fc) can effectively recognize CPV L4 in Western blot, ELISA, and immunofluorescence assays. Structural modeling, molecular docking, and molecular dynamics simulations supported stable binding interactions between Nb10/Nb10-Fc and VP2, providing a basis for subsequent epitope validation and antigenic variation monitoring. Overall, this study provides a valuable technical foundation for molecular surveillance of CPV-2c, optimization of diagnostic tools, and antibody engineering applications.

## Figures and Tables

**Table 1:** Sampling situation table.

Host Species	No. of Samples	No. of Positive Samples	Positive Rate (%)
Dog	30	8	26.67
Giant panda	2	0	0
Total	32	8	25

**Table 2:** Binding free energy decomposition.

Binding Free Energy Decomposition (kcal/mol)							
Complex	ΔGvdw	ΔGele	ΔGMM	ΔGpolar	ΔGapolar	ΔGsol	ΔG
CPV L4 VP2 + Nb10	−67.38 ± 0.22	−214.63 ± 1.16	−282.01 ± 1.21	254.97 ± 1.12	−9.41 ± 0.03	245.56 ± 1.10	−36.46 ± 0.42
CPV L4 VP2 + Nb10-Fc	−76.94 ± 0.27	−533.89 ± 2.26	−610.83 ± 2.33	587.57 ± 2.25	−11.84 ± 0.05	575.73 ± 2.22	−35.11 ± 0.44

ΔGvdw: Van der Waals energy; ΔGele: electrostatic energy; ΔGMM: molecular mechanics free energy; ΔGpolar: polar solvation free energy; ΔGapolar: non-polar solvation free energy; ΔGsol: solvation free energy.

## Data Availability

The datasets generated and/or analyzed during the current study are available from the corresponding author on reasonable request.

## Supplementary Materials

The following supporting information can be downloaded at https://www.mdpi.com/article/10.3390/ani16091402/s1, Figure S1: Residue binding free energy decomposition analysis results; Table S1: Virus sequences and GenBank accession numbers used in this study.
