# Supplementary material for: Molecular Characterization of Representative CPV-2c Isolates and Establishment of VP2-Targeted Nanobody-Based Immunodetection Tools
Source: Animals (Basel). 2026 May 3;16(9):1402. doi: 10.3390/ani16091402 (PMC13163062; doi:10.3390/ani16091402)
Supplement: Supplementary file 1 [file animals-16-01402-s001.zip › Supplementary Materials.pdf]

Supplementary Materials

Supplementary Table S1.

Serial Number	Virus Nomenclature	GenBank Accessions
1	CPV-L4	PX843427
2	CPV-L1	PX843424
3	CPV-L2	PX843425
4	CPV-L3	PX843426
5	CPV-L5	PX843428
6	CPV-L6	PX843429
7	CPV-L7	PX843430
8	CPV-L8	PX843431
9	CPV-2	M19296.1
10	CPV-2	MN451655.1
11	CPV-2	EU659116
12	CPV-2	MN451665
13	CPV-2a	MN451676
14	CPV-2a	MN451675
15	CPV-2a	JQ268283
16	CPV-2a	MN451674
17	CPV-2b	DQ340409
18	CPV-2b	AY742934
19	CPV-2c	OK094444.1
20	CPV-2c	MW650830.1
21	CPV-2c	MW811188.1
22	CPV-2c	ON322797.1
23	CPV-2c	OR399582.1
24	CPV-2c	MK518015.1
25	CPV-2c	MH476583
26	CPV-2c	MF001435.1
27	CPV-2c	LC216904.1
28	CPV-2c	MK895489
29	CPV-2c	KM457110.1
30	CPV-2c	FJ222821.1
31	FPV	EU659111.1
32	FPV	EU659112
33	CPV-2C	PP862237.1
34	CPV-2C	PP862238.1
35	CPV-2C	PP862239.1
36	CPV-2C	PP862240.1

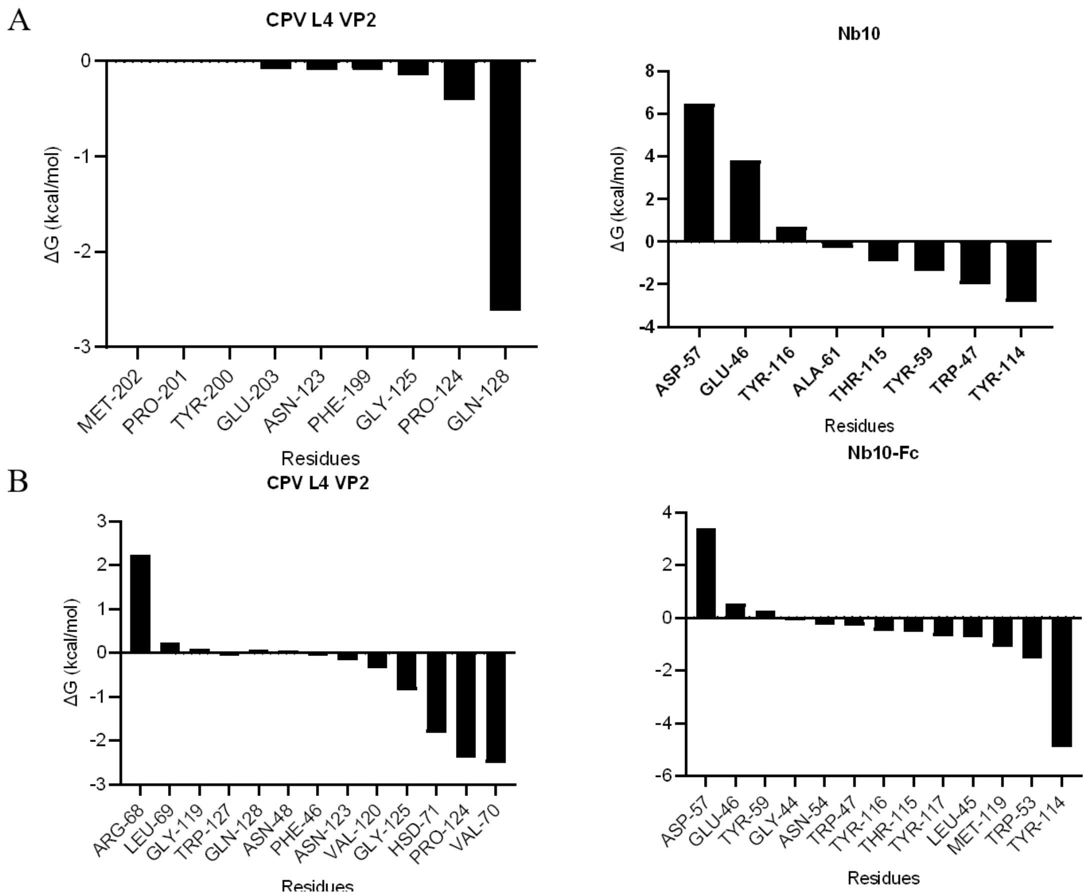

Supplementary Figure S1. Residue binding free energy decomposition analysis results
